# The complete mitochondrial genome of *Bathygobius cocosensis* (Perciformes, Gobiidae)

**DOI:** 10.1080/23802359.2018.1437824

**Published:** 2018-02-12

**Authors:** Jennifer L. Evans, Joshua A. Thia, Cynthia Riginos, James P. Hereward

**Affiliations:** School of Biological Sciences, The University of Queensland, Brisbane, Australia

**Keywords:** Mitogenome, Gobiidae, *Bathygobius cocosensis*

## Abstract

In this study, we sequenced the full mitochondrial genome of *Bathygobius cocosensis*, an abundant intertidal fish species, which may provide insights into the evolutionary genetics of chaotic genetic patchiness and range expansion in marine systems. The mitochondrial genome is 16,692 bp, and contains 13 protein-coding genes along with 22 tRNA and 2 rRNA genes and a D-loop region, arranged similarly to other Gobiidae species. A Bayesian phylogeny of Gobiidae species indicates close relationships to the genus *Glossogobius*. The *B. cocosensis* mitochondrial genome is now available through GenBank (Accession = MG704838).

Distributed throughout the Indo-Pacific, *Bathygobius cocosensis* is an abundant intertidal fish species that exhibits a bentho-pelagic life history; adults have high site fidelity (Malard et al. 2016), preceded by a planktonic larval phase of approximately 30 days (Thia, unpublished). Such life history facilitates long distance larval dispersal between distinct benthic populations of juveniles and adults. This, combined with their abundance along Australian coastlines and predictable yearly recruitment (Griffiths 2003a, 2003b) makes them an ideal species for the investigation of evolutionary processes underlying chaotic genetic patchiness and range expansion (Feary et al. 2013). Genomic resources, however, must be developed to support such investigation. In this study, we sequence the full mitogenome of *B. cocosensis*.

An individual fish was collected from Point Cartwright (Queensland, Australia), and DNA was extracted from fin tissue using the Omega E-Z 96 Tissue DNA Kit (Omega Bio-tek, Norcross, GA), following the ‘Tissues and Mouse Tail’ protocol. The specimen is stored at the University of Queensland (Brisbane, Australia). A genomic sequencing library was prepared using the NebNext Ultra DNA kit (New England Biolabs, Ipswich, MA) with an insert size of 340 bp. Sequencing (PE150 Illumina) was completed by Novogene (Beijing, China). Assembly was conducted in Geneious v10.2.3 (http://www.geneious.com, Kearse et al. 2012) using the mitochondrial genome of *Oxyurichthys formosanus* (Accession = NC_020345.1) as a reference to isolate mitochondrial DNA. Iterative remapping of reads onto contiguous sequences from this initial mapping stage was performed, and a consensus produced from a circular alignment of extended contiguous sequences. Annotation was completed by realigning the consensus *B. cocosensis* mitogenome to *O. formosanus*, transferring annotations, and manually inspecting sequences to check for abnormalities. A Bayesian phylogenetic tree (MrBayes (Huelsenbeck and Ronquist 2001)) was produced under the GTR + I + G model identified by jmodeltest2 (Darriba et al. 2012). This phylogeny was based on a MAFFT (Katoh and Standley 2013) alignment of coding sequences from 40 Gobiidae species’ mitochondrial genomes from GenBank and the new *B. cocosensis* mitochondrial genome. *Ecsenius bicolour* (Perciformes, Blennidae) was used as an outgroup.

The mitochondrial genome of *B. cocosensis* is 16,692 bp long, with a GC content of 48.9%. It contains 13 protein-coding genes, 22 tRNA genes, 2 rRNA genes, and a D-loop region. Structurally, it is very similar to other Gobiidae mitogenomes. Phylogenetic relationships based on mitochondrial coding sequences of the 41 Gobiidae species and the blenny *E. bicolour* (see Figure 1) indicates an unresolved relationship between *E. bicolour* and *Eleotris acanthopoma*. *Bathygobius cocosensis* is most closely related to the genus *Glossogobius*, within a clade including *O. formosanus*. The overall phylogenetic relationships are consistent with previous studies utilising partial and complete mitochondrial alignments within Gobiidae (Liu et al. 2013; Jin et al. 2015). The complete mitochondrial genome of *B. cocosensis* (Accession = MG704838) will allow further development of *B. cocosensis* as a model species for investigating evolutionary processes in marine systems.

**Figure 1.**
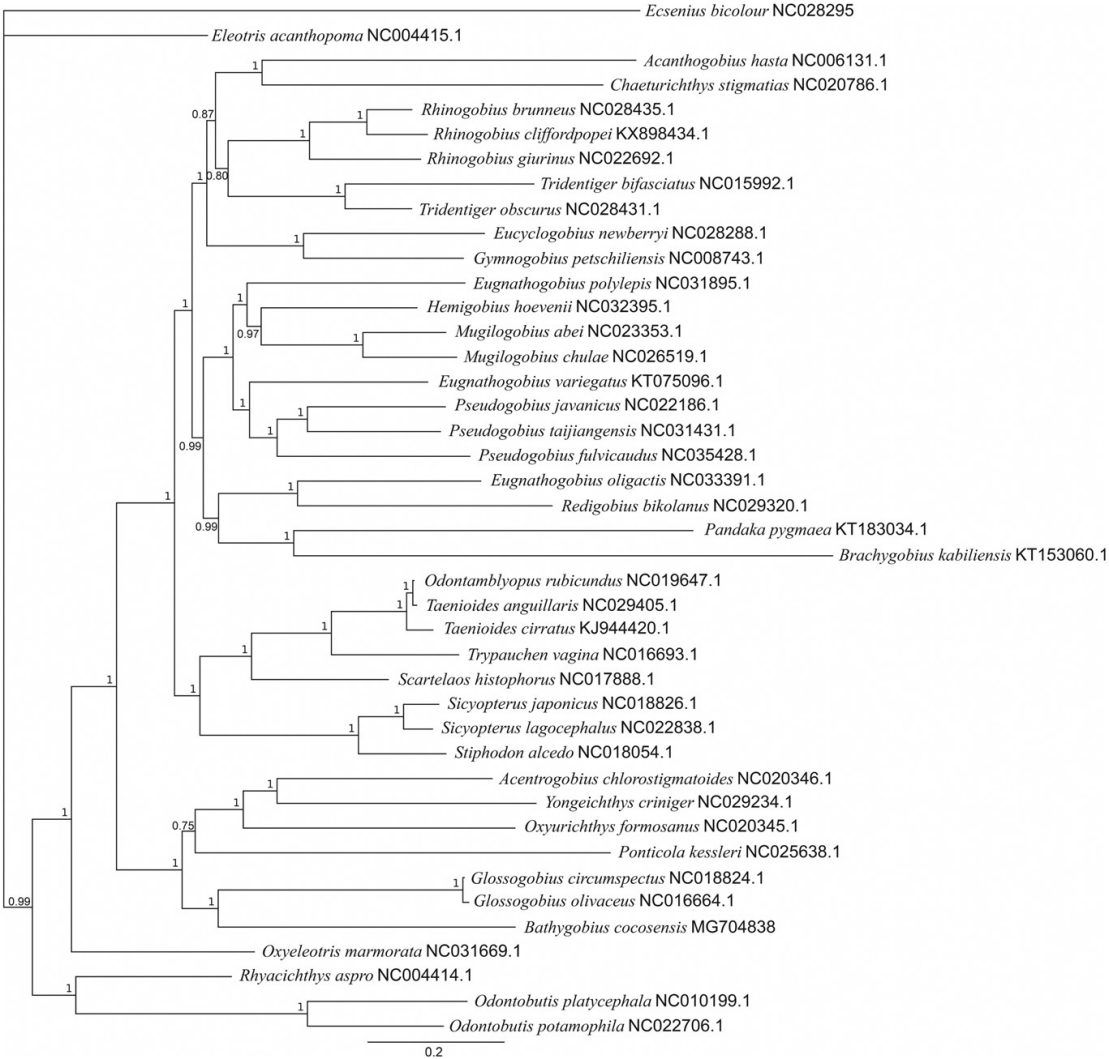
Phylogeny of 41 Gobiidae species produced through MrBayes under a GTR + I + G model, with *E. bicolour* (Perciformes, Blenniidae) as an outgroup, based on coding sequences of mitogenomes. Node labels indicate posterior probability.
